# Lipid Profile and Correlation to Cardiac Risk Factors and Cardiovascular Function in Type 1 Adolescent Diabetics from a Developing Country

**DOI:** 10.1155/2014/513460

**Published:** 2014-05-12

**Authors:** Aashima Dabas, Sangeeta Yadav, V. K. Gupta

**Affiliations:** ^1^Department of Paediatrics, Maulana Azad Medical College and Associated Lok Nayak Hospital, Bahadur Shah Zafar Marg, New Delhi 110002, India; ^2^Department of Biochemistry, G. B. Pant Hospital, Jawahar Lal Nehru Marg, New Delhi 110002, India

## Abstract

*Objective*. The adverse role of dyslipidemia in predicting cardiovascular outcomes has not been elucidated extensively among type 1 diabetics in the literature. *Methods*. We assessed dyslipidemia and its correlation to other cardiac risk factors in adolescents with type 1 diabetes. Total thirty type 1 adolescent diabetics were evaluated for their metabolic profile, including serum lipids and echocardiography was performed. *Results*. The average age of the cohort was 14.3 ± 3.09 yr with disease duration of 5.35 ± 2.94 yr. The mean HbA1C was 8.01%. The mean serum cholesterol, LDL, HDL, and triglyceride were normal. Serum cholesterol was high in patients with longer disease duration (*P* = 0.011, *r* = 0.41), high systolic blood pressure (*P* = 0.04, *r* = 0.32), and elevated HbA1C > 8% (*P* = 0.038, *r* = 0.33). Higher lipid values were associated with poorer carotid artery distensibility (*P* > 0.05) and higher carotid artery intimomedial thickness (cIMT) (*P* < 0.05 for cholesterol and LDL). Hyperglycemia adversely affected ejection fractions, though serum lipids did not show any significant effect on left ventricular parameters. Conclusions. Dyslipidemia and hyperglycemia can serve as biomarkers for cardiovascular dysfunction in at-risk adolescents with type 1 diabetes. Carotid artery parameters are adjunctive tools which may be affected early in the course of macrovascular disease.

## 1. Introduction

Early atherosclerotic lesions presenting as fatty streaks in blood vessels in childhood can progress in adolescence in the presence of risk factors like hyperlipidemia, hypertension, and diabetes mellitus (DM) [1]. There is enough evidence to support hyperglycemia as a risk factor for cardiovascular disease (CVD) in type 1 diabetes mellitus (T1DM). Hyperglycemia causes nonenzymatic glycation of proteins leading to formation of advanced glycation end products (AGEs) which are thought to be implicated in both microvascular and macrovascular complications of diabetes. They promote cross linking of proteins which can manifest as decreased cardiac compliance [2]. However, the role of lipids in causation of CVD has not been established in young adolescent diabetics. The prevalence of hyperlipidemia in T1DM is approximately 20–40% [3]. There is enhanced foam cell formation and an increase in the formation of low density lipoprotein cholesterol (LDL-C) dienes, which increase the susceptibility of LDL for oxidation and predispose for atherosclerosis [1, 4]. Chronic hyperglycemia induces a state of oxidative stress which in turn promotes low density lipoprotein (LDL) oxidation and a decrease in nitric oxide availability. Diabetes also promotes the expression of E-selectin and vascular cell adhesion molecule (VCAM) by the endothelial cells. These promote leucocyte adhesion to the vessels which accumulate lipids and release proinflammatory cytokines, thus promoting plaque formation [3, 5].

Hypertension acts as an independent cardiac risk factor and is postulated to promote smooth muscle proliferation that can increase intimomedial thickness (IMT) and left ventricle mass. It also adversely affects diastolic heart functions [3, 6].

Early changes of atherosclerosis include increased arterial stiffness and increased arterial IMT. These are followed by cardiac dysfunction and left ventricular hypertrophy. The arterial distensibility is postulated to be a more sensitive marker of atherosclerosis than cIMT as it measures endothelial dysfunction [5, 6]. There is limited data on noninvasive evaluation of cardiovascular functions in diabetic children. The incidence of early cardiovascular complications in T1DM has not been elucidated in the setting of a developing country.

We thus undertook a study to assess the metabolic control in young adolescent diabetics. The cardiovascular status of these patients was assessed using echocardiography and risk factors for the same were evaluated, with emphasis on lipid profile.

## 2. Methods

The study was conducted at the pediatric endocrinology clinic of a referral tertiary care hospital.

### 2.1. Sample Characteristics

All adolescent (10–18 years) type 1 diabetic patients who had regular follow-up at the endocrine clinic were assessed for enrolment in the study. They were diagnosed based on clinical and laboratory criteria; facilities for diagnosing islet cell antibodies were not available. All patients were screened for celiac disease by serum tissue transglutaminase levels and none had elevated titres. The thyroid function tests and anti-thyroid peroxidase antibodies measured at their initial diagnosis were also normal in all patients. The subjects were on premeal insulin bolus regimen (short and long acting insulin). Out of forty five such subjects, thirty consented and were evaluated. Patients were excluded if there was:

- any history of hypertension or intake of antihypertensive or lipid lowering medication,
- any history of substance abuse,
- any co morbid coexistent chronic disease,
- history of recent hospitalization/illness in the last month; such patients were evaluated after an interval of three months of apparent wellness.

The study design was prospective cross sectional. The study was approved by the institutional ethical committee. Informed consent was taken from the parents/guardians.

### 2.2. Clinical Evaluation

A general physical examination was performed and weight and height recorded. Body mass index (BMI) was calculated as weight (kg)/height^2^ (m)^2^. The BMI readings were interpreted using World Health Organization (WHO) charts and recorded to nearest percentile [7]. Baseline blood pressure (BP) was recorded using a standard sphygmomanometer in the supine position after a ten-minute rest period. An average of two readings was noted during any given measurement. The BP readings were interpreted against height and age adjusted BP centiles and a value ≥ 95th centile was considered abnormal if measured at ≥3 separate occasions [8]. Mean Bp was calculated as [(2 × DBP) + SBP]/3 [8].

### 2.3. Laboratory Evaluation

Both fasting and postprandial blood samples were collected by venipuncture in appropriate BD vacutainers using sterile technique. Fasting and postprandial blood glucose values were measured by the glucose oxidase method. Glycated hemoglobin (HbA1C) and lipid profile were measured on fasting blood samples. HbA1C was assessed using immunoturbidimetric method which measured the absorbance of the glycosylated hemoglobin fraction and total hemoglobin fraction at 415 nm. The lipid profile (total cholesterol, serum triglyceride, and serum high density lipoprotein-cholesterol (HDL-C)) was measured using standard methods on an autoanalyzer. Total cholesterol and triglycerides were measured using enzymatic colorimetric method. HDL-C was estimated by an automated direct assay method. LDL-C was calculated by Friedewald's formula [9]. 

### 2.4. Cardiovascular Function Evaluation

This was assessed using high resolution ultrasound scanner (AGILENT SONOS 4500 ULTRASOUND MACHINE) which was connected to computer software and obtained images were stored for future reference. The prerequisites for echocardiography measurements included fasting for 8–12 hours (to avoid changes in flow mediated arterial dilatation by substances like caffeine, high fat) and rest for 15 minutes [10]. All parameters were evaluated by a single experienced vascular sonographer who was blinded to the metabolic profile of the patients.

#### 2.4.1. Arterial Functions

The patients were placed supine with neck in slight hyperextension. The common carotid artery (below the carotid bulb and 1 cm proximal to bifurcation) was scanned on B mode (realtime) and Doppler imaging using a 7–12 MHz linear array transducer [1, 11]. Both right and left common carotid artery were evaluated and mean of three different recordings of both sides was taken as common final value.

*(1) Physiological Changes.* Flow mediated dilatation of the carotid artery (endothelium independent) was done to assess its distensibility. Distensibility is a measure of luminal diameter change in vessel with change in blood pressure [12]. Exogenous nitric oxide donor, for example, high dose nitroglycerine (NTG) tablet (0.4 mg sublingual) was given to obtain a vasodilator response. Peak vasodilatation occurs 3-4 minutes after drug administration during which blood pressure was monitored. Further evaluation was terminated on a patient if he developed hypotension or bradycardia. Images of the vessel before and after the drug administration were recorded and difference in diameter (distensibility) was noted.

*A2. Anatomical Changes.* Carotid intimal-medial thickness (cIMT) was assessed by measuring the near and far wall of the artery at two separate angles—anterior oblique and lateral. cIMT was measured as the difference between two echogenic lines of the vessel wall. The first line is the luminal-intimal interface while the second is collagen containing upper layer of adventitia [13, 14]. The normal limit for cIMT is arbitrary and is influenced by age, gender, and population. It is thus interpreted in terms of increased risk rather than statistical distribution; however a value of >1 mm is definitely abnormal [15].

#### 2.4.2. Cardiac Functions

The cardiac parameters (*M*-mode measurements) were recorded using the standard parasternal long axis view just below the tip of the mitral leaflet using 5 MHz phased array scanner [16]. The following parameters were recorded: left ventricular systolic and end diastolic internal dimensions (LVIDs, LVIDd), thickness of interventricular septum (IVS), fractional shortening at systole and diastole (FSs, FSd), and ejection fraction (EF) [17]. The apical four-chamber view was obtained for diastolic function analysis which included measurement of peak mitral inflow velocity at early diastole and at late diastole. Left ventricular inflow signals were obtained in the pulse mode by placing the sample volume between the mitral leaflets and adjusting the position until the highest peaks of diastolic velocity were obtained. The peak early diastolic velocity (*E*) and peak late diastolic velocity (*A*) were measured and ratio (*E*/*A*) was determined [18–20].

### 2.5. Statistical Analysis

The results were analyzed using appropriate statistical tests on SPSS software. Quantitative data was expressed as mean ± 2 SD. Statistical significance of quantitative variables between different categories was analyzed using *t*-test. Pearson's correlation coefficient/Spearman's rank coefficient (*r*) was used to indicate significant linear relationship among quantitative variables and regression analysis was done. A *P* value <0.05 was considered as significant. Any *P* value <0.001 was taken as highly significant.

## 3. Results 

A total of thirty diabetic patients were included in the study and all tolerated the study procedure well. There was an equal gender distribution with 15 boys and 15 girls. The mean age of patients was 14.3 ± 3.09 years. The body mass index (BMI) was in the range of 13.3–24 kg/m^2^ (mean 17.1 ± 2.9 kg/m^2^, at 25th centile of WHO chart). The average duration of the disease was 5.35 ± 2.94 years; two patients had diabetes for >10 years. The mean insulin dose was 1.1 U/kg/day. The average BP was 111.4 ± 12.52 mmHg systolic/70.48 ± 9.16 mmHg diastolic. Two patients were hypertensive when interpreted as per age and height chart [8]. The measured laboratory and echocardiography data have been summarized in Table 1. The observed HbA1C range was 4–13.1% (mean = 8.01 ± 2.19; normal range = 6–8%). The normal range of the lipid parameters was cholesterol, 150–200 mg/dL; triglyceride, 60–150 mg/dL; LDL, <100 mg/dL, as per kit inserts. The mean HDL was less than the reference of 40–60 mg/dL.

### 3.1. Lipids and Traditional Cardiac Risk Factors

**Figure 1:**
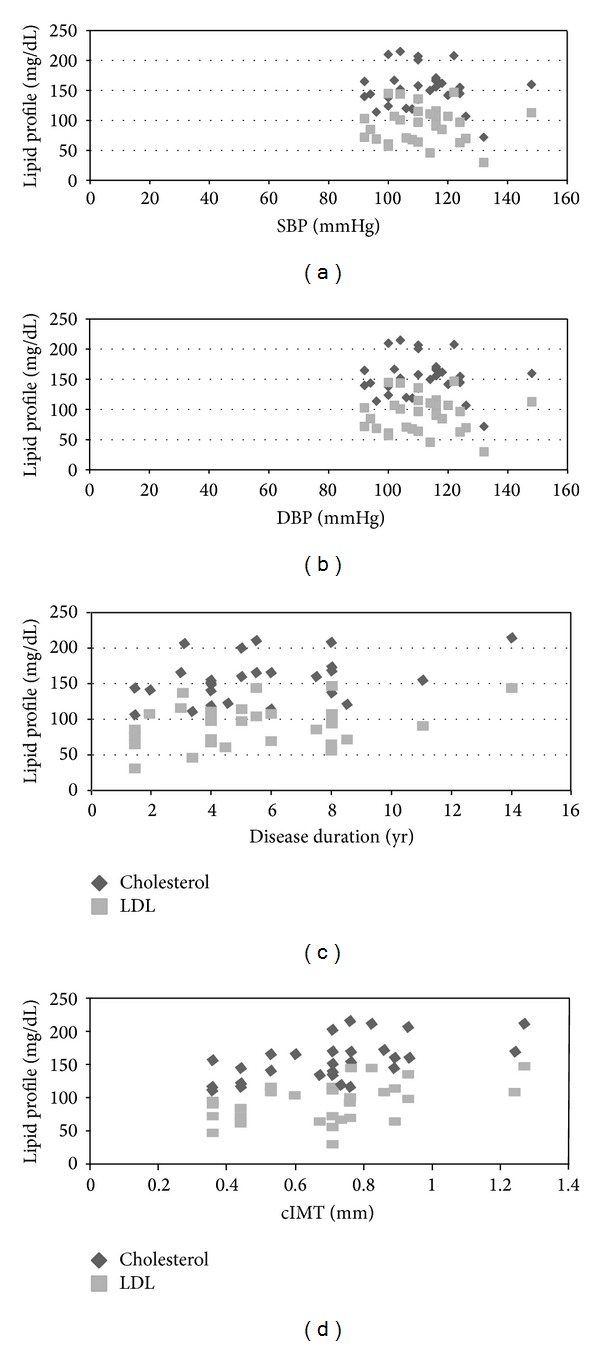
(a) Correlation of systolic BP with serum cholesterol and LDL. (b) Correlation of diastolic BP (DBP) with serum cholesterol and serum LDL. (c) Correlation of disease duration with serum cholesterol and serum LDL. (d) Correlation of cIMT with serum cholesterol and serum LDL. *(Each pair of points represents single subject).

None of the lipid parameters had any significant correlation to age (*P* > 0.05). Among lipids, serum cholesterol had a significant positive correlation with SBP (*P* = 0.04, *r* = 0.32; Figure 1(a)) and weaker with DBP (*P* > 0.05; *r* = 0.26; Figure 1(b)); the rest of lipid parameters had weaker correlation with BP. A significant correlation was established between serum cholesterol and LDL with duration of disease (*P* = 0.011, *r* = 0.41, *P* = 0.049, *r* = 0.30, Figure 1(c)) unlike the rest of lipid parameters.

### 3.2. Cardiovascular Parameters

There was no difference in arterial distensibility or cIMT when compared with age or gender (*P* > 0.05).  Table 2 depicts the relation of different cardiovascular variables evaluated on echocardiography with metabolic parameters. On multivariate analysis, the arterial distensibility remained unaffected by changes in blood pressure, duration of disease, fasting sugar, HbA1C, and lipid profile (*P* > 0.05). However, there was a significant inverse relation between postprandial sugar and arterial distensibility (*P* = 0.05; *r* = − 0.43). Both serum cholesterol and LDL had a significant correlation with cIMT, (*P* = 0.002; *r* = 0.48 and *P* = 0.017; *r* = 0.44, resp. (Figure 1(d)). Serum triglycerides and HDL did not show any correlation to arterial parameters. There was no observed significance between cIMT and duration of disease or fasting/postprandial sugar (>0.05). Both SBP and DBP were significantly related to cIMT (*P* = 0.02; *r* = 0.37 and *P* = 0.005; *r* = 0.46, resp.). Serum lipid parameters did not affect left ventricular functions in our cohort (*P* > 0.05).

### 3.3. Glycemic Control and Analysis

Patients with a poorer sugar profile (HbA1C ≥ 8%) had longer disease duration (*P* = 0.009; *r* = 0.44), higher DBP (*P* = 0.047, *r* = 0.37), higher serum cholesterol (*P* = 0.011; *r* = 0.41), and higher serum LDL (*P* = 0.55; *r* = 0.29). The arterial distensibility was better with good glycemic (HbA1C < 8%) values unlike those with higher HbA1C, though the result was not significant (*P* = 0.75). The mean cIMT was significantly higher in patients with higher HbA1C; *P* = 0.019; *r* = 0.43. There was a significant difference with ejection fractions measured during systole (74.4 ± 7.2% in good control and 68.18 ± 6.3% in poor control); *P* = 0.02. The ejection fraction at diastole was also significantly higher in those with good control (69.1 ± 7.1%) as compared to those with poor control (64.07 ± 5.52); *P* = 0.03. Rest of the LV parameters did not establish a significant relation with HbA1C (*P* > 0.05).

LV internal diameters were directly influenced with cIMT values (*P* = 0.048 in systole and *P* = 0.042 in diastole). Five patients in our cohort had increased interventricular septal thickness; two of them also had high cIMT (>0.8 mm); (*P* = 0.013). The mitral inflow velocity in late diastole (*A*) was significantly associated with carotid artery distensibility (*P* < 0.01) and with cIMT (*P* = 0.002).

## 4. Discussion

The present cohort of adolescent diabetics had a near normal metabolic control with mean lipid profile being normal. Patients with high serum lipids (especially cholesterol and LDL) were at high risk of CVD as they recorded higher blood pressure and had longer disease duration, poorer glycemic control (HbA1C > 8%), and deranged arterial parameters as measured on echocardiography.

Diabetic patients are prone to develop dyslipidemia (quantitative and qualitative) with reported prevalence of 24–40% [21–24]. Diabetics with suboptimal HbA1C may have deranged lipid values versus those with optimal HbA1C [25]. Serum HDL levels are generally optimum in T1DM and do not play a causative role in CVD [26, 27]. The mean lipids were higher in our patients with poorer glycemic control. Though our patients with longer disease duration had poorer glycemic control and higher serum cholesterol, they did not manifest any significant changes in the carotid artery parameters, similar to data reported by Gunczler et al. [28]. Conversely few other authors have identified early cardiac changes in the presence of longer diabetes duration [29, 30].

The coexistence of diabetes and hypertension has been considered as a major factor in the expression of the abnormalities in human diabetic myocardium [13, 15, 31]. Both serum cholesterol and glycemic control predicted higher systolic BP in our study. Abdelghaffar et al. and Carugo et al. have reported a similar difference in blood pressures of diabetics versus controls in their studies [32, 33]. However, Aepfelbacher et al. did not document any difference in BP of T1DM even after a documented improvement in HbA1C [34]. Hypertension emerged as a risk factor for increased cIMT in our cohort similar to prior conducted studies [35–38]. The American Diabetes Association (ADA) thus recommends blood pressure evaluation in all diabetics on follow-up visits for cardiovascular screening [39]. They have also defined optimal serum lipid levels in children with T1DM but the threshold to decide intervention is still under research [40].

BMI is a nonlipid cardiac risk factor for atherogenesis [41, 42] and is raised in T1DM [12, 32]. The BMI recorded in our cohort was low as poverty and lower socioeconomic status determined BMI in our setting.

There was no significant correlation between carotid artery distensibility and any of the lipid values, though serum cholesterol and LDL had significant relation to cIMT. Various study trials have reported hyperlipidemia to be associated with carotid atherosclerosis in T1DM [4, 32, 38, 43]; Järvisalo et al. concluded the role of both serum cholesterol and LDL levels in predicting arterial structural integrity in T1DM, even if they were present within the normal range in blood [1, 38]. However few like Peppa-Patrikiou et al. have reported inconclusive results [35]. These outcomes are variable probably because of various confounding factors like age, blood pressure, family history of dyslipidemia, disease duration, and HbA1C status.

Hyperglycemia was established as a cardiac risk factor in our study similar to earlier studies. Correlation of HbA1C was seen with arterial distensibility [12], cIMT [12, 32, 35, 38, 44], and few ventricular parameters [33, 34, 45–47] as described in past. In a recent publication by DCCT, cIMT has been established as a marker for atherosclerosis when associated with hyperglycemia [48]. However, there are few authors who have obtained normal cIMT [12, 28, 49] and normal LV functions in patients with T1DM [50].

Left ventricular changes are frequently reported in diabetes in existence of glucose intolerance [42]. There are limited available human studies where lipid values are correlated to cardiac functions in T1DM. There was no correlation between lipids and LV parameters in our study which was probably due to a short disease duration; as postulated earlier by Chen et al [51].

The study was one of the first pilot studies which assessed left ventricular functions in adolescent diabetics in relation to lipid profile. However the major limitation of our study was a small sample size and absence of healthy control population for comparison. The disease duration was also small to influence significant changes in LV functions. Future studies comparing diabetic population with healthy controls will be needed on a larger scale to reinforce the results of this first pilot study.

## 5. Conclusion

To summarize, both serum cholesterol and LDL were established as cardiac risk factors, in addition to HbA1C and blood pressure. Dyslipidemia can serve as early biomarker for cardiovascular dysfunction in adolescents with Type 1 Diabetes.

What is already known about this topic is as follows.

1. Type 1 diabetes is associated with cardiovascular morbidity.
2. Patients with type 1 diabetes need to be screened for dyslipidemia around puberty.

What this paper adds is as follows.

(1) Dyslipidemia may not be overt early in course of type 1 diabetes.
(2) Raised serum cholesterol and serum LDL can serve as biomarkers for cardiovascular morbidity in adolescents with type 1 diabetes.
(3) Echocardiography may be used as an adjunct to monitor cardiac status in diabetics with poor metabolic control to prevent complications.

## Figures and Tables

**Table 1:** Mean laboratory and echocardiographic parameters of study population.

Parameter	Mean ± S. D.
Fasting blood glucose	223.8 ± 108.8 mg/dL
Postmeal glucose (2 hours later)	267.9 ± 114.79 mg/dL
HbA1C	8.01%
S. Cholesterol	152.70 ± 33.5 mg/dL
S. Triglyceride	111.8 ± 49.61 mg/dL
S.HDL	38.31 ± 11.38 mg/dL
S.LDL	92.39 ± 29.73 mg/dL
Carotid distensibility	0.097 ± 0.064 mm.
cIMT	0.698 ± 0.23 mm
LVID [s]/LVID [d]	3.65 ± 1.05 cm/4.69 ± 1.32 cm
Interventricular septal thickness	8.49 ± 1.20 mm
EF [s]/EF [d]	70.6 ± 7.3%/66.11 ± 6.6%
FS [s]/FS [d]	35.84 ± 3.6 cm/32.9 ± 3.42 cm
*E*/*A* ratio	1.25 ± 0.97

**Table 2:** Cardiovascular variables compared with metabolic parameters—showing significance values (*P*).

Echo parameter/metabolic parameter	HbA1C	Serum cholesterol	Serum triglyceride	Serum HDL	Serum LDL
Carotid distensibility	0.75	0.63	0.87	0.30	0.92
cIMT	0.02*	0.002*	0.06	0.10	0.017*
Ejection fraction (systole)	0.02*	0.197	0.142	0.463	0.501
Ejection fraction (diastole)	0.03*	0.204	0.090	0.590	0.513

*Significant value.
